# Effects of Adaptive Servo-Ventilation on Nocturnal Ventricular Arrhythmia in Heart Failure Patients With Reduced Ejection Fraction and Central Sleep Apnea–An Analysis From the SERVE-HF Major Substudy

**DOI:** 10.3389/fcvm.2022.896917

**Published:** 2022-06-20

**Authors:** Christoph Fisser, Lara Gall, Jannis Bureck, Victoria Vaas, Jörg Priefert, Sabine Fredersdorf, Florian Zeman, Dominik Linz, Holger Woehrle, Renaud Tamisier, Helmut Teschler, Martin R. Cowie, Michael Arzt

**Affiliations:** ^1^Department of Internal Medicine II, University Medical Centre Regensburg, Regensburg, Germany; ^2^Center for Clinical Studies, University Medical Centre Regensburg, Regensburg, Germany; ^3^Department of Cardiology, Maastricht University Medical Centre and Cardiovascular Research Institute Maastricht, Maastricht, Netherlands; ^4^Department of Cardiology, Radboud University Medical Centre, Nijmegen, Netherlands; ^5^Department of Biomedical Sciences, Faculty of Health and Medical Sciences, University of Copenhagen, Copenhagen, Denmark; ^6^Centre for Heart Rhythm Disorders, Royal Adelaide Hospital, University of Adelaide, Adelaide, SA, Australia; ^7^Sleep and Ventilation Center Blaubeuren, Lung Center Ulm, Ulm, Germany; ^8^Univ. Grenoble Alpes, INSERM 1300, HP2, Grenoble, France; ^9^Service Hospitalo-Universitaire Pneumologie et Physiologie, Pole Thorax et Vaisseaux, CHU de Grenoble Alpes, Grenoble, France; ^10^Department of Pneumology, AFPR, Ruhrlandklinik, West German Lung Center, University Hospital Essen, Essen, Germany; ^11^Royal Brompton Hospital and School of Cardiovascular Medicine and Sciences, Faculty of Life Sciences and Medicine, King's College London, London, United Kingdom

**Keywords:** heart failure, central sleep apnoea, adaptive servo-ventilation, ventricular arrhythmias, SERVE-HF

## Abstract

**Background:**

The SERVE-HF trial investigated the effect of treating central sleep apnoea (CSA) with adaptive servo-ventilation (ASV) in patients with heart failure with reduced ejection fraction (HFrEF).

**Objective:**

The aim of the present ancillary analysis of the SERVE-HF major substudy (NCT01164592) was to assess the effects of ASV on the burden of nocturnal ventricular arrhythmias as one possible mechanism for sudden cardiac death in ASV-treated patients with HFrEF and CSA.

**Methods:**

Three hundred twelve patients were randomized in the SERVE-HF major substudy [no treatment of CSA (control) vs. ASV]. Polysomnography including nocturnal ECG fulfilling technical requirements was performed at baseline, and at 3 and 12 months. Premature ventricular complexes (events/h of total recording time) and non-sustained ventricular tachycardia were assessed. Linear mixed models and generalized linear mixed models were used to analyse differences between the control and ASV groups, and changes over time.

**Results:**

From baseline to 3- and 12-month follow-up, respectively, the number of premature ventricular complexes (control: median 19.7, 19.0 and 19.0; ASV: 29.1, 29.0 and 26.0 events/h; *p* = 0.800) and the occurrence of ≥1 non-sustained ventricular tachycardia/night (control: 18, 25, and 18% of patients; ASV: 24, 16, and 24% of patients; *p* = 0.095) were similar in the control and ASV groups.

**Conclusion:**

Addition of ASV to guideline-based medical management had no significant effect on nocturnal ventricular ectopy or tachyarrhythmia over a period of 12 months in alive patients with HFrEF and CSA. Findings do not further support the hypothesis that ASV may lead to sudden cardiac death by triggering ventricular tachyarrhythmia.

## Introduction

High ventricular arrhythmia burden or higher grade ventricular arrhythmias such as frequent premature ventricular complexes (PVCs >30/h) or non-sustained ventricular tachycardia (NSVT) are associated with impaired left ventricular function and increased mortality in patients with heart failure and reduced left ventricular ejection fraction (HFrEF) (1–5). Decreasing the burden of PVCs can prevent the development of PVC-induced cardiomyopathy, which may improve prognosis in patients with HFrEF (4). Furthermore, shortly coupled PVCs may also trigger ventricular arrhythmias contributing to sudden cardiac death.

Sleep-disordered breathing (SDB), either obstructive (OSA) or central (CSA) sleep apnoea, is a common comorbidity in HFrEF, occurring in up to 50% of patients (6). Several studies suggest that SDB-related conditions may contribute to ventricular arrhythmogenesis in HFrEF by various mechanisms (7). In a previous small randomized controlled trial in patients with HFrEF, treatment of co-existing OSA with continuous positive airway pressure (CPAP) reduced the frequency of PVCs during sleep (8). In another randomized controlled pilot trial in patients with HFrEF, treatment of CSA, coexisting CSA-OSA or OSA using flow-triggered adaptive servo-ventilation (ASV) showed a trend toward a reduction of nocturnal PVCs and NSVT (9).

The Treatment of Sleep-Disordered Breathing with Predominant Central Sleep Apnea by Adaptive Servo Ventilation in Patients with Heart Failure (SERVE-HF) trial is the largest randomized trial to investigate the effect of treating CSA with ASV compared with guideline-based medical treatment alone (control) in patients with HFrEF and CSA (10). Unexpectedly, rates of all-cause and cardiovascular mortality were significantly higher in patients randomized to the ASV vs. control group (10). It has been hypothesized that ASV may increase the burden of ventricular arrhythmia triggering sudden death as one potential reason for the observed increased all-cause and cardiovascular mortality in the ASV group (11). Several explanations as to how ASV may trigger ventricular arrhythmias-such as rapid changes in blood gases, pH and potassium levels, in addition to effects on venous return and transmural wall tension by applied positive airway pressure-have been proposed (12).

Therefore, the aim of the present ancillary analysis of the SERVE-HF major substudy (NCT01164592) (13) was to assess the effects of ASV on the burden of nocturnal ventricular arrhythmias (PVCs and NSVT) in ASV-treated patients with HFrEF and CSA.

## Materials and Methods

### Study Design and Participants

Of 91 centres participating in SERVE-HF (10), seven contributed patients to the SERVE-HF major substudy (NCT01164592) including assessment of SDB with full PSG with ECG (13). Inclusion and exclusion criteria have been previously reported in detail (10, 13). Briefly, patients were aged ≥22 years and had symptomatic chronic HF (New York Heart Association [NYHA] class III or IV, or class II with ≥1 HF-related hospitalization in the previous 24 months) and reduced left ventricular ejection fraction (LVEF ≤45%) (13). All received stable, guideline-based medical treatment for HF (13, 14). Anti-arrhythmic drugs (chiefly amiodarone) were left to the discretion of the investigators. With respect to SDB, individuals had predominant CSA (13). Study exclusion criteria were amyloidosis, hypertrophic cardiomyopathy, and diuretic dosage more than doubled within the 4 weeks prior to randomization (13).

For the present non-prespecified ancillary analysis, additional exclusion criteria were insufficient electrocardiogram (ECG) data such as technical interference, artifacts, unclear rhythm or no consensus in the endpoint adjudication committee, as published previously (15).

CSA was defined as an apnoea-hypopnoea index (AHI) >15/h with ≥50% central events and a central AHI ≥10/h, derived from polysomnography (PSG) and based on total recording time, documented within 4 weeks of randomization, with flow measurement performed using a nasal cannula (10). All PSGs were centrally scored in a blinded fashion (HP2 Sleep CoreLab, Alpes University, Grenoble, France) by two scorers (and a third if discrepancy occurred) according to AASM criteria (16, 17).

The substudy protocol was approved by the appropriate local or regional ethics committees [110420d/110420f (Adelaide), 2011-06-303 (Brisbane), HREC-D 153-11 (Melbourne), HPH323 (Perth), HREC/11/WMEAD/124 (Sydney), 27PZT/2012 (Czech Republic), H-D-2008-034 (Denmark), 293/13/03/01/2011 (Finland), 08-RESM-1 (France), 010/1553 (Germany), AA11 (The Netherlands), 2009/2083/REK vest (Norway), dnr M38-08 (Sweden), Rif CE 2581 (Switzerland), 08/H1307/41(UK)] (11). The trial was conducted according to Good Clinical Practice and the Principles of the Declaration of Helsinki 2002. All participants gave written informed consent.

### Study Intervention and Assessment

SERVE-HF participants were randomly assigned to receive guideline-based medical treatment alone (control) or guideline-based medical treatment with ASV (Auto Set CS, ResMed). Full details of ASV titration and settings have been reported previously (10). Substudy evaluations such as PSG were performed at baseline, and at 3 and 12 months after randomization. The substudy was completed when all 312 patients had been followed for 12 months.

### ECG Measurements–Cardiac Arrhythmias

ECG data were derived from full overnight PSG as described previously (15). The nocturnal holter ECG was monitored from a single precordial lead with sampling frequency of 250 Hz, acceptable for analysis of ventricular arrhythmias (18, 19).

Data sets were visually analysed by two trained investigators (LG, JB) according to standard definitions (9, 19, 20) with interobserver variability for PVCs comparable with previous important analyses of nocturnal ECGs in cohorts with PSG (19–21) [intraclass correlation coefficient (95% confidence interval) for a random sample of 20 sleep studies: 0.90 (0.75–0.96), *p* < 0.001] (15). Investigators were blinded with respect to clinical data and intervention, and only had access to the ECG channel and the pre-scored sleep stages of the PSG. Cardiac rhythm was analysed according to standard criteria (15, 22).

### Study Outcomes

The endpoints of this ancillary analysis of the SERVE-HF major substudy were changes in nocturnal ventricular arrhythmias, including PVCs/h, frequent PVCs (>30/h) and higher grade ventricular arrhythmias (e.g., ≥1 NSVT/night) between baseline and 12 months' follow-up in the ASV and control groups (15).

### Statistical Analysis

Categorical data are presented as absolute and relative frequencies and were compared using the Chi-Square Test of Independence. Normally and non-normally distributed quantitative data are presented as mean ± standard deviation and median [interquartile range (IQR)], respectively. Quantitative variables were compared between groups using either unpaired Student's *t*-test for normally distributed data or Mann-Whitney U-Test for non-normally distributed variables. Linear mixed models (LMM) based on ranks for continuous data and generalized linear mixed models (GLMM) for binary outcomes were used to analyse treatment effects on several clinical outcomes. Group, time and the group-time interaction term were added as factors and the baseline value of the variable of interest was added as covariate. Time was specified as a repeated-measures factor with an unstructured covariance matrix for the LMMs and compound symmetry covariance matrix for the GLMMs. Further models were calculated by adjusting additionally for LVEF, and use of angiotensin-converting enzyme inhibitors, angiotensin receptor blockers and β-receptor blockers.

All reported *p*-values are two-sided, and a *p*-value of 0.05 was the threshold for statistical significance. Data entry and calculation were performed with the software package SPSS 26.0 (Chicago, EUA). Linear and generalized mixed models were calculated using SAS 9.4 (SAS Institute, 2013, Cary NC) and the procedures *proc mixed* and *proc glimmix*.

## Results

### Study Population

**Figure 1:**
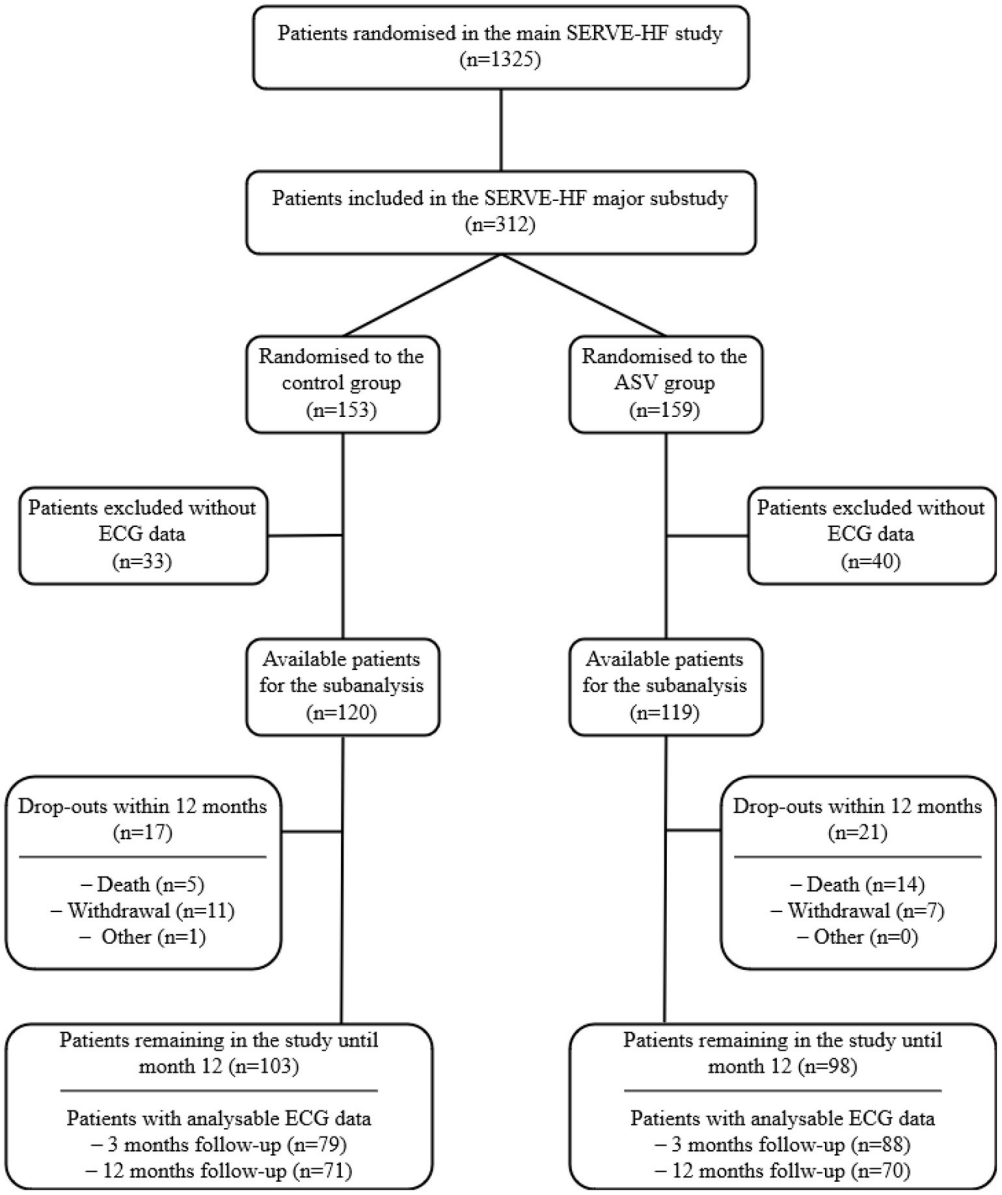
Flowchart for the ancillary analysis of the effects of treatment with adaptive servo-ventilation (ASV) of participants from the SERVE-HF major substudy. ECG, electrocardiogram.

Of the 312 participants in the SERVE-HF major substudy, 239 had analysable ECG data that fulfilled the technical requirements (120 in the control group and 119 in the ASV group at baseline) (Figure 1). Patients had a mean age of 69 years and were mainly male with an average LVEF of 33%, with the etiology of heart failure most commonly classified as due to ischaemic heart disease. More than 50% had a cardiac implantable electronic device. However, neither at baseline nor at follow-up, a systematic assessment of cardiac arrhythmias is available in these. There were no significant differences between the groups at baseline (Table 1; e-Table 1 in Supplementary Material). Nocturnal respiratory baseline characteristics were similar in the control and the ASV groups with respect to severity of sleep apnoea (AHI), type of sleep apnoea (cAHI/AHI), percent time spent in Cheyne Stokes Respiration (CSR), oxygen desaturation index and time with oxygen saturation <90% (Table 2). In the ASV group the AHI was reduced by 78% to 9 events/h both at 3 and 12 months (e-Tables 2, 3 in Supplementary Material). Mean ASV usage at baseline, at 3 and 12 months was 6.3 ± 2.0, 4.5 ± 2.4, and 4.3 ± 2.5 h, respectively. Follow-up ECG data at 3 and 12 months were available in 167 (control: 79, ASV: 88) and 141 (control: 71, ASV: 70) patients, respectively.

**Table 1:** Patient demographic and clinical characteristics at baseline.

	**Control (*n* = 120)**	**ASV (*n* = 119)**	***p*-value**
Age, years	69 ± 10	69 ± 10	0.793
Male, *n* (%)	109 (91)	109 (92)	0.835
Body mass index, kg/m^2a, b^	29.5 ± 5.2	29.3 ± 5.0	0.735
Diabetes mellitus, *n* (%)^a, b^	53 (44)	56 (48)	0.649
Blood pressure, mmHg			
Systolic^b, c^	123 ± 18	124 ± 19	0.680
Diastolic^b, c^	73 ± 12	74 ± 11	0.684
Nocturnal Holter ECG heart	69 ± 12	68 ± 12	0.598
rate, beats/min			
QRS duration, ms^a, d^	133 ± 37	134 ± 37	0.710
QRS >120 ms, *n* (%)^a, d^	63 (53)	62 (53)	0.940
Bundle branch block^a, b^, *n* (%)			0.635
Right	10 (8)	7 (6)	
Left	31 (26)	28 (24)	
Other	22 (18)	29 (25)	
NYHA class, *n* (%)			0.406
I	0 (0)	1 (1)	
II	26 (22)	31 (26)	
III	94 (78)	87 (73)	
IV	0 (0)	0 (0)	
LVEF*, %	34 ± 8	32 ± 8	0.133
HF etiology, *n* (%)			0.132
Ischaemic	76 (63)	65 (55)	
Other	44 (37)	54 (45)	
Any implanted device, *n* (%)	65 (54)	68 (57)	0.750
Non-CRT pacemaker	5 (4)	7 (6)	
ICD	36 (30)	30 (25)	
CRT-P	1 (1)	1 (1)	
CRT-D	23 (19)	30 (25)	
Creatinine^†^, mg/dL^e, f^	1.4 ± 0.7	1.3 ± 0.5	0.289
Cardiac medication, *n* (%)			
ACEI or ARB	116 (97)	108 (91)	0.060
β-receptor blocker	112 (93)	105 (88)	0.173
Aldosterone antagonist	70 (58)	72 (61)	0.733
Diuretic	105 (88)	96 (81)	0.149
Cardiac glycoside	21 (18)	30 (25)	0.146
Anti-arrhythmics	16 (13)	17 (14)	0.831

*Data are expressed as number of patients (%), or mean ± standard deviation. ACEI, angiotensin-converting enzyme inhibitor; ARB, angiotensin receptor blocker; ASV, adaptive servo-ventilation; CRT, cardiac resynchronization therapy; CRT-D, CRT with defibrillator; CRT-P, CRT with pacemaker; eGFR, estimated glomerular filtration rate; HF, heart failure; ICD, implantable cardioverter-defibrillator; LVEF, left ventricular ejection fraction; NYHA, New York Heart Association*.

*^†^Locally measured data after enrolment in the trial*.

^*^Locally measured data, up to ≤ 3 months prior to the trial.

*^a^Data available for 120/120 control group patients*.

*^b^Data available for 118/119 ASV group patients*.

*^c^Data available for 119/120 control group patients*.

*^d^Data available for 117/119 ASV group patients*.

*^e^Data available for 116/120 control group patients*.

*^f^Data available for 113/119 ASV group patients*.

**Table 2:** Respiratory characteristics at baseline.

**Characteristics**	**Control** **(*n* = 120)**	**ASV** **(*n* = 119)**	***p*-value**
AHI, events/h TST	39.2 ± 14.6	38.8 ± 14.6	0.821
Apnoea index, events/h TST	23.7 ± 18.3	22.6 ± 17.7	0.620
cAHI, % of AHI	79.4 ± 16.5	77.5 ± 16.0	0.381
Oxygen desaturation index^‡^	34.6 ± 19.8	33.6 ± 18.4	0.696
events/h TST			
Oxygen saturation, %			
Mean	93 ± 2	93 ± 2	0.726
Minimum	81 ± 8	81 ± 7	0.938
Time with oxygen	18 [4; 66]	23 [5; 62]	0.796
saturation <90%, min			
CSR, *n* (%)	109 (91)	109 (92)	0.835
CSR proportion of TRT*			0.692
<20%	27 (25)	30 (28)	
20–49	41 (38)	44 (40)	
>49	41 (38)	35 (32)	

*Data are expressed as number of patients (%), mean ± standard deviation, or median [interquartile range]. AHI, apnoea-hypopnoea index; ASV, adaptive servo-ventilation; TST, total sleeping time; TRT, total recording time; cAHI, central apnoea-hypopnoea index; CSR, Cheyne Stokes respiration*

^*^Values are rounded.

*Data were missing for the following characteristics: time with an oxygen saturation of <90% for 1 in the ASV-group*.

*The oxygen desaturation index is the number of times that the blood oxygen level drops by ≥3 percentage points from baseline per hour of recording time*.

### Ventricular Arrhythmias

**Figure 2:**
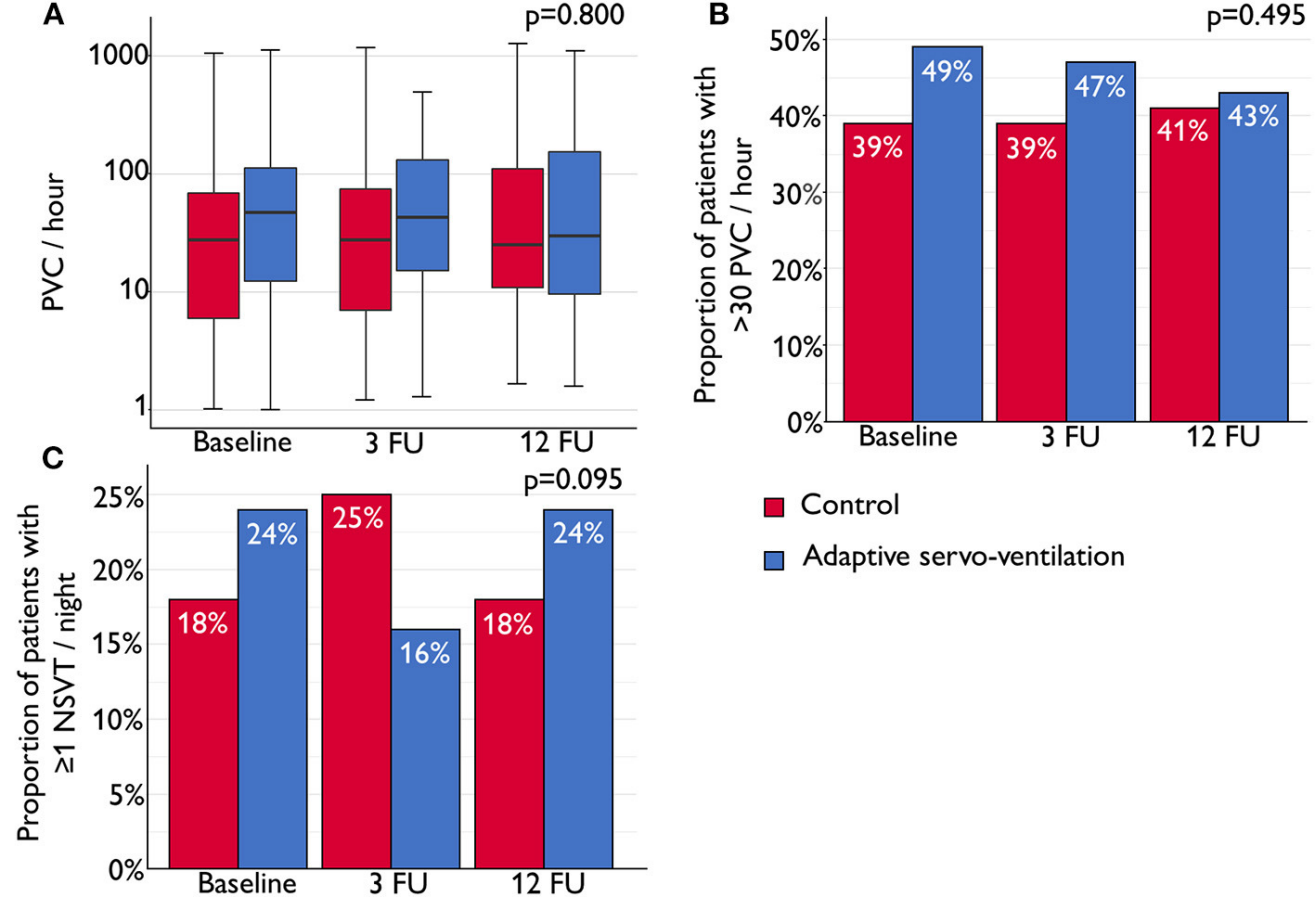
**(A)** Box plot shows the median number of nocturnal premature ventricular complexes (PVCs) per hour of total recording time (horizontal line) with interquartile range (IQR) at baseline, 3-month follow-up (3 FU) and 12-month FU (12 FU). Whiskers show maximum/minimum value still within 1.5*IQR of upper/lower quartile. **(B)** Bar chart showing the proportion of patients with >30 nocturnal PVC per hour at baseline, 3 FU and 12 FU. **(C)** Bar chart showing the proportion of patients with ≥1 nocturnal non-sustained ventricular tachycardia (NSVT) event per night in the control and adaptive servo-ventilation (ASV) groups at baseline, 3 FU and 12 FU. Linear mixed models (LMM) based on ranks for continuous data **(A)** and generalized linear mixed models (GLMM) for binary outcomes **(B,C)** were used to analyse treatment effects for differences between the control and ASV groups and for changes over time. Group, time and the interaction term (group*time) were added as factors and the baseline value of the variable of interest was added as covariate.

There were no significant differences between the ASV and control groups with respect to the number of nocturnal PVC/h, and the proportion of patients with >30 PVC/h and ≥1 NSVT/night (Figure 2). Comparing the rate of nocturnal cardiac arrhythmias between groups using (generalized) linear mixed models, no significant differences with respect to the group, time or group^*^time (interaction) were seen (Table 3). Findings were similar after further adjustment for numerical differences at baseline (including LVEF) and use of angiotensin-converting enzyme inhibitors, angiotensin receptor blockers and β-receptor blockers (Table 3).

**Table 3:** Nocturnal ventricular arrhythmias at baseline, and 3- and 12-month follow-up.

	**Control**		**ASV**		***p*** **-values**	
	***N***	**Median [IQR] or *n* (%)**	***N***	**Median [IQR] or *n* (%)**	**Model 1**	**Model 2**
**Nocturnal ventricular arrhythmias, events per hour recording time**						
PVC/h at baseline	120	19.7 [3; 58]	119	29.1 [3; 95]	G: *p* = 0.413	G: *p* = 0.414
PVC/h at 3-month follow-up	79	19.0 [2; 63]	88	29.0 [2; 101]	T: *p* = 0.516	T: *p* = 0.482
PVC/h at 12-month follow-up	71	19.0 [5; 84]	70	26.0 [6; 118]	I: *p* = 0.800	I: *p* = 0.800
**High burden of nocturnal ventricular arrhythmias**, ***n*****, (%)**						
>30 PVC/h at baseline	120	47 (39)	119	58 (49)	G: *p* = 0.866	G: *p* = 0.883
>30 PVC/h at 3-month follow-up	79	31 (39)	88	41 (47)	T: *p* = 0.519	T: *p* = 0.550
>30 PVC/h at 12-month follow-up	71	29 (41)	70	30 (43)	I: *p* = 0.495	I: *p* = 0.511
**Nocturnal high grade ventricular arrhythmias**, ***n*****, (%)**						
≥1 NSVT at baseline	120	21 (18)	119	29 (24)	G: *p* = 0.473	G: *p* = 0.564
≥1 NSVT at 3-month follow-up	79	3 (25)	88	5 (16)	T: *p* = 0.860	T: *p* = 0.798
≥1 NSVT at 12-month follow-up	71	13 (18)	70	17 (24)	I: *p* = 0.095	I: *p* = 0.083

*Data are expressed as median [interquartile range (IQR)] or number of patients (%). P-values (G, group; T, Time; I, Interaction Group*

^*^Time) correspond to linear mixed models based on ranks for the first endpoint and on generalized linear mixed models for the second and third endpoint.

*Model 1: adjusted for baseline value of outcome variable; Model 2: adjusted for baseline value of outcome variable, left ventricular ejection fraction, intake of angiotensin converting enzyme inhibitors and β-receptor blockers*.

*ASV, adaptive servo-ventilation; NSVT, non-sustained ventricular tachycardia; PVC, premature ventricular complex*.

### Subgroup Analyses

**Figure 3:**
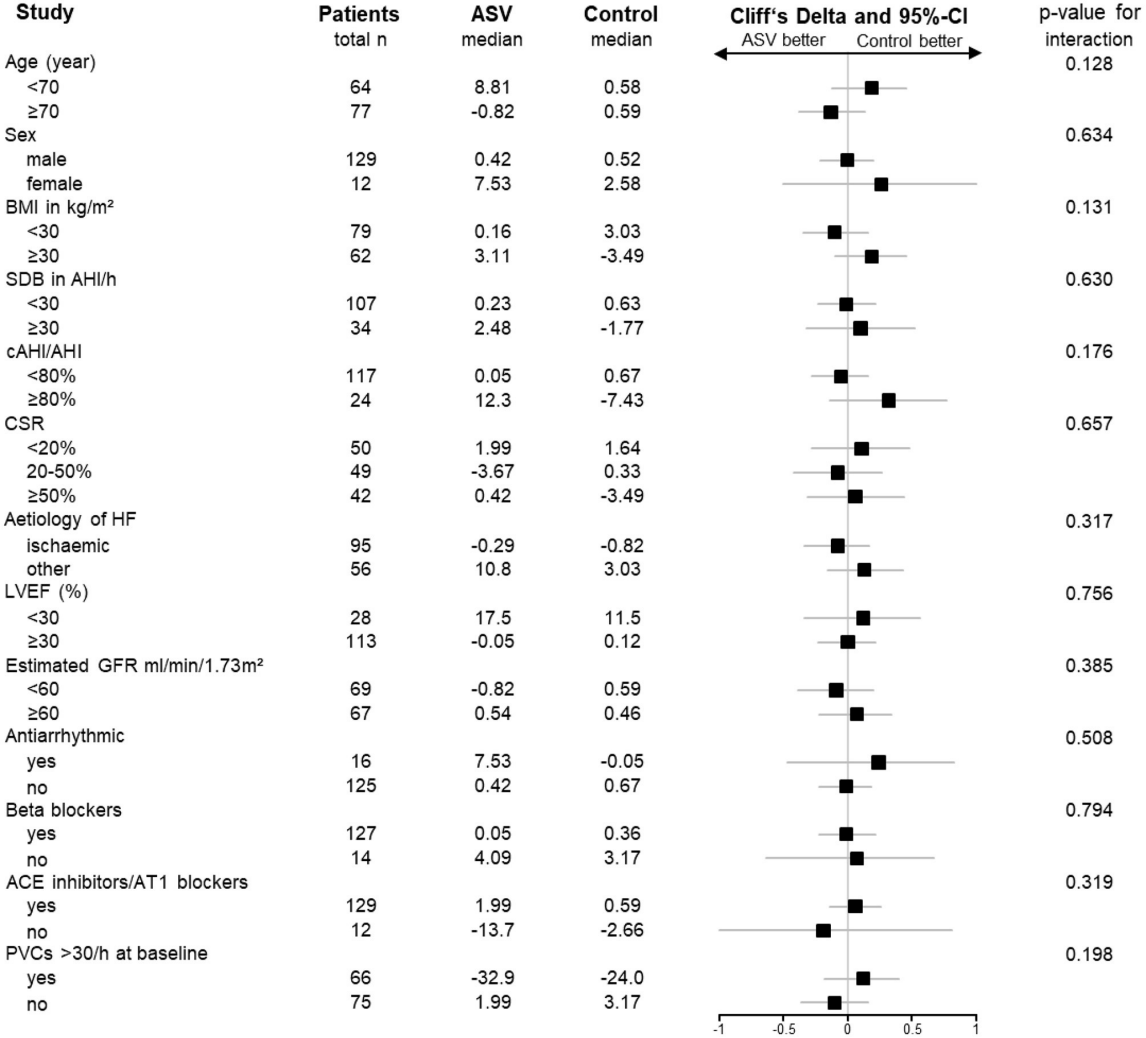
Forrest plot of treatment effects on the number of nocturnal premature ventricular complexes (PVCs) per hour in predefined patient subgroups based on the parent SERVE-HF study (10). Treatment effects are presented as Cliff's Delta with the corresponding 95% confidence interval (CI). *P*-values are derived from the interaction term (group*characteristic) from the linear mixed-effects model based on ranks. ACE, angiotensin converting enzyme; AHI, apnoea-hypopnoea index; AT1, angiotensin II type 1 receptor; BMI, body mass index; cAHI, central apnoea-hypopnea-index; CSR, Cheyne-Stokes-Respiration; GFR, glomerular filtration rate; HF, heart failure; LVEF, left ventricular ejection fraction; NYHA, New York Heart Association; PVC, premature ventricular complex; SDB, sleep-disordered breathing.

Across, and within, all pre-specified subgroups, no different treatment effects (control vs. ASV) on nocturnal PVC/h were observed (all interaction terms *p* > 0.05), including in subgroups based on baseline LVEF (<30% vs. ≥30%), the proportion of CSR (<20% vs. ≥20%) and baseline PVCs (>30/h vs. ≤30/h) (Figure 3; e-Table 4 in Supplementary Material). In the ASV group, the PVC burden at 3 months was similar in those patients with AHI <15/h (*n* = 69) compared to those patients with AHI ≥15/h (*n* = 19) at 3 months (29.7 [3.3; 116] vs. 15.1 [0.7; 55.5], *p* = 0.271).

## Discussion

This study provides unique insights into the observed effects of ASV on nocturnal ventricular arrhythmias in patients with HFrEF and CSA. First, the change in number of nocturnal PVC/h from baseline to 3- and 12-month follow-up was similar in the control and ASV groups. Second, ASV had no significant effect on the proportion of patients with a high burden of nocturnal ventricular arrhythmias (>30 PVC/h) or higher grade nocturnal ventricular arrhythmias (≥1 NSVT/night). Third, all prespecified subgroup analyses did not favour either the control or the ASV group with respect to changes of nocturnal PVC/h over time. This included important subgroups included patients with low LVEF (<30%) (10, 11), low proportion of CSR <20% (10), and high burden of ventricular arrhythmias (1).

In an ancillary analysis of the SERVE-HF major substudy, burden of nocturnal PVC >30/h was observed in 44% of patients with HFrEF and CSA (15). A higher cardiovascular mortality in the ASV group of the SERVE-HF parent study has been proposed to result from sudden cardiac death, presumably due to cardiac arrhythmias (11). To date, randomized controlled trials evaluating the effect of positive airway pressure treatment on ventricular arrhythmias in patients with HFrEF and SDB are scarce and included only a few (or no) patients with CSA. This is important because the pathophysiological impact of obstructive and central apnoeas and PVCs is clearly different (e.g., the timing of PVCs in relation to the apnoea) (7, 23).

In a small randomized pilot trial (*n* = 20) including HFrEF patients with severe SDB (mean LVEF 32% and AHI 49/h; with CSA, coexisting CSA and OSA, or OSA) ASV also did not impact the PVC burden compared with the control group (9). Another small randomized trial (*n* = 18) analysed the effect of CPAP on PVC/h in OSA patients (mean AHI 29/h) with HFrEF (mean LVEF 28%) (8). Compared with the control group not receiving CPAP therapy, CPAP was associated with a 58% reduction in the number of PVC/h within 1 month of starting treatment (8). The present study is the first randomized controlled trial to evaluate the effects of ASV on the change in PVC burden over time, and the rates of high PVC burden and higher-grade ventricular arrhythmias in HFrEF patients with CSA and did not find any significant differences between the ASV and control groups. Similar to our results, data from a substudy of the Cardiovascular Improvements With Minute Ventilation-Targeted ASV Therapy in Heart Failure (CAT-HF) trial in hospitalized patients with heart failure with either OSA and/or CSA (AHI >15/h) and with implanted cardioverter-defibrillator (ICD) or cardiac resynchronization therapy with defibrillator (*n* = 46) reported that the rate of ventricular tachycardia and ventricular fibrillation was similar between groups treated with ASV added to guideline-based medical therapy compared with guideline-based medical therapy only (24).

In addition to the overall analysis, the current study evaluated all endpoints in important patient subgroups. This included patients with a baseline CSR proportion ≥20%, a subgroup in which those in the ASV vs. control group in the parent SERVE-HF study had higher rates of all-cause death or life-saving cardiovascular intervention plus unplanned hospitalization for worsening chronic heart failure (10). Furthermore, CSR ≥20% has been reported to be an independent predictor of PVC/h and the burden of PVC was 86% higher during periods of CSR compared to periods without CSR (15). Another subgroup analysis was based on baseline LVEF because subgroup analysis of the SERVE-HF parent study revealed that ASV was associated with higher cardiovascular mortality in those with baseline LVEF ≤30% (10, 11). In addition, lower LVEF is associated with an increased risk for ventricular arrhythmias in patients without SDB (25), which might also be the case in patients with SDB. Finally, patients were divided into subgroups based on baseline burden of ventricular arrhythmias (PVCs >30 vs. ≤30/h) because PVCs >30/h have been associated with a 2.6-fold higher mortality in the general population (1, 5, 25), but this had not yet been evaluated in patients with HFrEF and CSA. For example, Ryan et al. (8) analysed patients with OSA, the majority of whom had PVCs >30/h, and reported that the rate of this arrhythmia decreased from 170 PVC/h to 70 PVC/h in the group treated with CPAP therapy group compared to an increase from 84/h to 101/h in the control group. The hypothesis was that the effect of treating SDB on ventricular arrhythmias might be more pronounced in those with a high burden of PVCs. However, the present subanalyses did not show any modification of the effect of ASV on PVC burden in these patient subgroups.

The results of our analysis neither further support the hypothesis that CSA contributes to ventricular arrhythmogenesis nor that ASV increases sudden cardiac death by triggering ventricular tachyarrhythmia. In addition, ICD discharge data from the parent SERVE-HF study does not support a tachyarrhythmia mechanism for potentially increased sudden cardiac death (hazard ratio for appropriate shock in the ASV vs. control arm was 0.71 [95% confidence interval 0.48–1.04]; *p* = 0.08, with rates of 0.024 vs. 0.033 events/year) (10).

This suggests that mechanisms other than tachyarrhythmia (e.g., asystole or electromechanical dissociation that may result from hypoxia and hypercapnia, arousals and sympathetic activation) may be the underlying mechanism for the increase in cardiovascular mortality reported in patients randomized to the ASV vs. control group in the SERVE-HF trial (12). However, in the CANPAP trial suppression of CSA by means of CPAP had no effect on mortality compared to the control group (26). Similarly, in the Bad Oeynhausen prospective ASV registry, ASV therapy was not associated with increased mortality in patients with CSA and HFrEF (27). Therefore, the effect of ASV needs to be investigated in future studies.

The findings of this analysis have to be interpreted in the light of the following limitations. Nocturnal ECG data were obtained in the seven centers participating in SERVE-HF major substudy (13) during one night at baseline, 3 and 12 months only and 24-hour Holter ECG data were not available (13). However, the sampling frequency of the ECG lead during PSG is acceptable for analysis of ventricular and supraventricular arrhythmias (15, 19), and the PVC burden in patients with HFrEF and SDB is similar during the day and at night (28). In addition, cardiovascular deaths in SERVE-HF were distributed throughout the 24-h period. The impact of PVC suppression remains controversial. On the one hand treatment with antiarrhythmic medication have been reported to result in higher mortality (29). On the other hand, in patients with left ventricular systolic dysfunction, ablation of frequent PVCs induces a significant improvement in functional, structural, and neurohormonal status and a sustained reduction in the baseline PVC burden is associated with a lower risk of cardiac mortality, cardiac transplantation, or hospitalization for heart failure (30).

In addition, we cannot rule out the possibility that ASV may have triggered ventricular arrhythmias in some cases during the daytime or in patients who died before the follow-up sleep studies. However, this was not systematically assessed in the SERVE-HF major substudy (13).

## Conclusion

In conclusion, addition of ASV to guideline-based medical management had no significant effect on nocturnal ventricular ectopy or tachyarrhythmia over a period of 12 months in alive patients with HFrEF and CSA. Findings do not further support the hypothesis that ASV may lead to sudden cardiac death by triggering ventricular tachyarrhythmia.

## Data Availability Statement

The original contributions presented in the study are included in the article/Supplementary Material, further inquiries can be directed to the corresponding author/s.

## Ethics Statement

The substudy protocol was approved by the Appropriate Local or Regional Ethics Committees [110420d/110420f (Adelaide), 2011-06-303 (Brisbane), HREC-D 153-11 (Melbourne), HPH323 (Perth), HREC/11/WMEAD/124 (Sydney), 27PZT/2012 (Czech Republic), H-D-2008-034 (Denmark), 293/13/03/01/2011 (Finland), 08-RESM-1 (France), 010/1553 (Germany), AA11 (The Netherlands), 2009/2083/REK vest (Norway), dnr M38-08 (Sweden), Rif CE 2581 (Switzerland), 08/H1307/41(UK)]. The trial was conducted according to Good Clinical Practice and the Principles of the Declaration of Helsinki 2002. All participants gave written informed consent. The patients/participants provided their written informed consent to participate in this study.

## Author Contributions

MA was responsible for conceiving and designing the study and its hypotheses, acquiring study funding, collecting, analysing and interpreting the data, and writing and revising the manuscript prior to submission. CF and MA are the guarantor of the content of the manuscript and were involved in the collection, analysis and interpretation of data, and were responsible for drafting and revising the manuscript prior to submission. LG, JB, VV, JP, SF, DL, HW, RT, HT, and MC were involved in the collection and interpretation of data and critical revision of the manuscript prior to submission. FZ was involved in data analysis and critical revision of the manuscript prior to submission. All authors contributed to the article and approved the submitted version.

## Funding

The SERVE-HF trial was supported by ResMed and by grants from the National Institute for Health Research (NIHR) Cardiovascular and Respiratory Biomedical Research Units (general award to the hospital; MC). Representatives and scientists from the ResMed participated in the study including design and data collection. This ancillary analysis was supported by ResMed (MA) and by a grant from the German Heart Foundation/German Foundation of Heart Research (F/15/20; CF).

## Conflict of Interest

CF reports receiving support from the German Heart Foundation/German Foundation of Heart Research. VV received grant support from the German Society of Sleep Medicine. On behalf of DL, the University of Maastricht has received lecture fees and/or consulting fees and/or research grants from Bayer, LivaNova, ResMed and Respicardia. HW was a former employee of ResMed during the SERVE-HF study. RT has received consulting fees from Agiradom (Healthcare provider) and grant support from ResMed. MC has received consulting fees from ResMed and Respicardia, and grant support through his Institution from ResMed, Bayer and Abbott. HT has received consulting fees, grant support, and hardware and software for the development of devices from ResMed. MA has received consulting fees from ResMed, Philips Respironics, Boehringer-Ingelheim, NRI, Novartis, JAZZ Pharmaceuticals, Bayer, Inspire and Bresotec,and grant support from ResMed Foundation, Philips Respironics and the Else-Kroener Fresenius Foundation (2018_A159) outside the submitted work. The remaining authors declare that the research was conducted in the absence of any commercial or financial relationships that could be construed as a potential conflict of interest.

## Publisher's Note

All claims expressed in this article are solely those of the authors and do not necessarily represent those of their affiliated organizations, or those of the publisher, the editors and the reviewers. Any product that may be evaluated in this article, or claim that may be made by its manufacturer, is not guaranteed or endorsed by the publisher.
