# Supplementary material for: Effects of Adaptive Servo-Ventilation on Nocturnal Ventricular Arrhythmia in Heart Failure Patients With Reduced Ejection Fraction and Central Sleep Apnea–An Analysis From the SERVE-HF Major Substudy
Source: Front Cardiovasc Med. 2022 Jun 20;9:896917. doi: 10.3389/fcvm.2022.896917 (PMC9252521; doi:10.3389/fcvm.2022.896917)
Supplement: Supplementary file 1 [file Data_Sheet_1.docx]

**Supplementary material**

This appendix is provided by the authors to give additional information about the research.

Supplement to:

**Effects of adaptive servo-ventilation on nocturnal ventricular arrhythmia in heart failure patients with reduced ejection fraction and central sleep apnea – an analysis from the SERVE-HF major substudy**

Christoph Fisser, MD^1^, Lara Gall^1^, Jannis Bureck^1^, Victoria Vaas^1^, Jörg Priefert, MD^1^, Sabine Fredersdorf, MD^1^, Florian Zeman, M. Sc.^2^, Dominik Linz, MD^3,4,5,6^, Holger Woehrle, MD^7^, Renaud Tamisier, MD^8,9^, Helmut Teschler, MD^10^, Martin R Cowie, MD^11^, Michael Arzt, MD^1^

^1^Department of Internal Medicine II, University Medical Centre Regensburg, Regensburg, Germany

^2^Center for Clinical Studies, University Medical Centre Regensburg, Regensburg, Germany

^3^Department of Cardiology, Maastricht University Medical Centre and Cardiovascular Research Institute Maastricht, Maastricht, the Netherlands

^4^Department of Cardiology, Radboud University Medical Centre, Nijmegen, the Netherlands

^5^Department of Biomedical Sciences, Faculty of Health and Medical Sciences, University of Copenhagen, Copenhagen, Denmark

^6^Centre for Heart Rhythm Disorders, Royal Adelaide Hospital, University of Adelaide, Adelaide, Australia

^7^Sleep and Ventilation Center Blaubeuren, Lung Center Ulm, Ulm, Germany

^8^Univ. Grenoble Alpes, Inserm 1300, HP2, Grenoble, France

^9^Service Hospitalo-Universitaire Pneumologie et Physiologie, Pole Thorax et Vaisseaux, CHU de Grenoble Alpes, Grenoble, France

^10^Department of Pneumology, AFPR, Ruhrlandklinik, West German Lung Center, University Hospital Essen, Essen, Germany

^11^Royal Brompton Hospital & School of Cardiovascular Medicine & Sciences, faculty of Life Sciences & Medicine, King’s College London, London, United Kingdom

**e-Table 1** Additional patient demographic and clinical characteristics at baseline

|  | **Control (n=120)** | **ASV (n=119)** |
| --- | --- | --- |
| Body weight, kg^a^ | 90.0±19.2 | 88.6±15.9 |
| Six-minute walk distance, m^b,c^ | 339±128 | 340±123 |
| Haematocrit^d,e^, % | 42±4 | 42±4 |
| Haemoglobin^d,e^, g/dL | 14±2 | 14±2 |
| eGFR, mL/min/1.73 m² ^f,g^ | 59±23 | 59±20 |
| Device implantation >6 months ago, n (%) | 61 (51) | 62 (52) |
| Country, n (%*) |  |  |
| Australia | 9 (8) | 14 (12) |
| Switzerland | 1 (1) | 1 (1) |
| Czech Republic | 4 (3) | 3 (3) |
| Germany | 99 (83) | 94 (79) |
| Finland | 1 (1) | 1 (1) |
| France | 4 (3) | 5 (4) |
| United Kingdom | 2 (2) | 1 (1) |

Data are expressed as number of patients (%), mean ± standard deviation, or median [interquartile range].

BNP, brain natriuretic peptide.

*Values are rounded.

^a^Data available for 118/119 ASV group patients

^b^Data available for 113/120 control group patients.

^c^Data available for 111/119 ASV group patients.

^d^Data available for 118/120 control group patients.

^e^Data available for 115/120 ASV group patients.

^f^Data available for 116/120 control group patients.

^g^Data available for 113/119 ASV group patients.

**e-Table 2** Respiratory characteristics at 3 months follow-up

| **Characteristics** | **ASV (n=119)** |
| --- | --- |
| AHI, events/h TST^a^ | 8.5±11.7 |
| Apnoea index, events/h TST^a^ | 2.9±8.3 |
| cAHI, % of AHI | 39.9±42.0 |
| Oxygen desaturation index^‡^, events/h TST^a^ | 9.8±11.5 |
| Oxygen saturation, %^a^ |  |
| Mean | 94±2 |
| Minimum | 86±4 |
| Time with oxygen saturation <90%, min^a^ | 2.0 [0.3; 11.1] |
| CSR, n (%)^a^ | 12 (10) |
| CSR proportion of TRT* |  |
| <20% | 6 (50) |
| 20–49 | 4 (33) |
| >49 | 2 (17) |

Data are expressed as number of patients (%), mean ± standard deviation, or median [interquartile range]. AHI, apnoea-hypopnoea index; ASV, adaptive servo-ventilation; TST, total sleeping time; TRT, total recording time; cAHI, central apnoea-hypopnoea index; CSR, Cheyne Stokes respiration

*Values are rounded.

Data were missing for the following characteristics: time with an oxygen saturation of less than 90% for 1 in the ASV- group

^‡^The oxygen desaturation index is the number of times that the blood oxygen level drops by ≥3 percentage points from baseline per hour of recording time.

^a^Data available for 106/119 ASV group patients.

^b^Data available for 92/119 ASV group patients.

**e-Table 3** Respiratory characteristics at 12 months follow-up

| **Characteristics** | **ASV (n=119)** |
| --- | --- |
| AHI, events/h TST^a^ | 8.6±13.1 |
| Apnoea index, events/h TST^a^ | 2.7±9.4 |
| cAHI, % of AHI^b^ | 46.7±45.4 |
| Oxygen desaturation index^‡^, events/h TST^a^ | 9.2±12.1 |
| Oxygen saturation, %^a^ |  |
| Mean | 94±2 |
| Minimum | 86±7 |
| Time with oxygen saturation <90%, min^a^ | 1.5 [0.0; 14.0] |
| CSR, n (%)^a^ | 12 (10) |
| CSR proportion of TRT* |  |
| <20% | 6 (50) |
| 20–49 | 2 (17) |
| >49 | 4 (33) |

Data are expressed as number of patients (%), mean ± standard deviation, or median [interquartile range]. AHI, apnoea-hypopnoea index; ASV, adaptive servo-ventilation; TST, total sleeping time; TRT, total recording time; cAHI, central apnoea-hypopnoea index; CSR, Cheyne Stokes respiration

*Values are rounded.

Data were missing for the following characteristics: time with an oxygen saturation of less than 90% for 1 in the ASV- group

^‡^The oxygen desaturation index is the number of times that the blood oxygen level drops by ≥3 percentage points from baseline per hour of recording time.

^a^Data available for 81/119 ASV group patients.

^b^Data available for 72/119 ASV group patients.

**e-Table 4** Treatment effects on the number of nocturnal premature ventricular complexes per hour in predefined patient subgroups

| **Subgroup** | **Patients, n** | **ASV** | **Control** | **Cliff’s Delta (95% CI)** | **p-value for interaction** |
| --- | --- | --- | --- | --- | --- |
|  | **Total n** | **Median of difference** | **Median of difference** | **Negative favors ASV** |  |
| Age (years) |  |  |  |  | 0.128 |
| <70 | 64 | 8.81 | 0.58 | 0.19 (–0.12, 0.46) |  |
| ≥70 | 77 | –0.82 | 0.59 | –0.13 (–0.38, 0.13) |  |
| Gender |  |  |  |  | 0.634 |
| Male | 129 | 0.42 | 0.52 | –0.002 (–0.21, 0.20) |  |
| female | 12 | 7.53 | 2.58 | 0.26 (–0.5, 1) |  |
| Body mass index in kg/m² |  |  |  |  | 0.131 |
| <30 | 79 | 0.16 | 3.03 | –0.10 (–0.35, 0.16) |  |
| ≥30 | 62 | 3.11 | –3.49 | 0.19 (–0.10, 0.46) |  |
| SDB (AHI events/hour) |  |  |  |  | 0.630 |
| <30 | 107 | 0.23 | 0.63 | –0.01 (–0.23, 0.22) |  |
| ≥30 | 34 | 2.48 | –1.77 | 0.10 (–0.32, 0.52) |  |
| cAHI/AHI |  |  |  |  | 0.176 |
| <80% | 117 | 0.05 | 0.67 | –0.05 (–0.28, 0.16) |  |
| ≥80% | 24 | 12.3 | –7.43 | 0.32 (–0.14, 0.77) |  |
| Cheyne Stokes Respiration |  |  |  |  | 0.657 |
| <20% | 50 | 1.99 | 1.64 | 0.08 (–0.24, 0.40) |  |
| 20-50% | 49 | –3.67 | 0.33 | –0.08 (–0.42, 0.27) |  |
| ≥50% | 42 | 0.42 | –3.49 | 0.06 (–0.31, 0.44) |  |
| Etiology of heart failure |  |  |  |  | 0.317 |
| Ischemic | 95 | –0.29 | –0.82 | –0.08 (–0.34, 0.17) |  |
| Other | 56 | 10.8 | 3.03 | 0.13 (–0.16, 0.43) |  |
| Left ventricular ejection fraction (%) |  |  |  |  | 0.756 |
| <30 | 28 | 17.5 | 11.5 | 0.12 (–0.34, 0.56) |  |
| ≥30 | 113 | –0.05 | 0.12 | 0.002 (–0.23, 0.22) |  |
| Estimated GFR |  |  |  |  | 0.385 |
| <60 | 69 | –0.82 | 0.59 | –0.09 (–0.39, 0.20) |  |
| ≥60 | 67 | 0.54 | 0.46 | 0.07 (–0.22, 0.34) |  |
| Antiarrhythmic |  |  |  |  | 0.508 |
| Yes | 16 | 7.53 | –0.05 | 0.24 (–0.47, 0.83) |  |
| no | 125 | 0.42 | 0.67 | –0.01 (–0.22, 0.18) |  |
| β–receptor blockers |  |  |  |  | 0.794 |
| Yes | 127 | 0.05 | 0.36 | –0.01 (–0.22, 0.22) |  |
| No | 14 | 4.09 | 3.17 | 0.07 (–0.64. 0.67) |  |
| ACE inhibitors/ AT1 blockers |  |  |  |  | 0.319 |
| Yes | 129 | 1.99 | 0.59 | 0.06 (–0.14, 0.26) |  |
| no | 12 | –13.7 | –2.66 | –0.19 (–1, 0.81) |  |
| PVCs >30/h at baseline |  |  |  |  | 0.198 |
| Yes | 66 | –32.9 | –24.0 | 0.12 (–0.18, 0.40) |  |
| no | 75 | 1.99 | 3.17 | –0.1 (–0.36, 0.16) |  |

ACE, angiotensin-converting enzyme; AHI, apnoea-hypopnoea index; AT1, angiotensin II type 1 receptor; BMI, body mass index; cAHI, central apnoea-hypopnoea index; CSR, Cheyne Stokes respiration; GFR, glomerular filtration rate; HF, heart failure; LVEF, left ventricular ejection fraction; NYHA, New York Heart Association; PVC, premature ventricular complex; SDB, sleep-disordered breathing.
